# FOXO1 Content Is Reduced in Cystic Fibrosis and Increases with IGF-I Treatment

**DOI:** 10.3390/ijms151018000

**Published:** 2014-10-08

**Authors:** Arianna Smerieri, Luisa Montanini, Luigi Maiuri, Sergio Bernasconi, Maria E. Street

**Affiliations:** 1 Department of Pediatrics, University Hospital of Parma, Via Gramsci 14, 43126 Parma, Italy; E-Mails: arianna.smerieri@unipr.it (A.S.); luisa.montanini@unipr.it (L.M.); SBernasconi@ao.pr.it (S.B.); 2 European Institute for Research in Cystic Fibrosis, San Raffaele Scientific Institute, Via Olgettina 60, 20132 Milan, Italy; E-Mail: maiuri@unina.it; 3 Institute of Pediatrics, University of Foggia, Via Pinto, 71100 Foggia, Italy

**Keywords:** cystic fibrosis-related diabetes, insulin, insulin resistance, IRS1, AKT, FOXO1, β2 arrestin, SOCS2, ERK1 and 2, IGF-I

## Abstract

Cystic fibrosis-related diabetes is to date the most frequent complication in cystic fibrosis (CF). The mechanisms underlying this condition are not well understood, and a possible role of insulin resistance is debated. We investigated insulin signal transduction in CF. Total insulin receptor, IRS1, p85 PI3K, and AKT contents were substantially normal in CF cells (CFBE41o-), whereas winged helix forkhead (FOX)O1 contents were reduced both in baseline conditions and after insulin stimulation. In addition, CF cells showed increased ERK1/2, and reduced β2 arrestin contents. No significant change in SOCS2 was observed. By using a CFTR inhibitor and siRNA, changes in FOXO1 were related to CFTR loss of function. In a CF-affected mouse model, FOXO1 content was reduced in the muscle while no significant difference was observed in liver and adipose tissue compared with wild-type. Insulin-like growth factor 1 (IGF-I) increased FOXO1 content *in vitro* and *in vivo* in muscle and adipose tissue. In conclusion; we present the first description of reduced FOXO1 content in CF, which is compatible with reduced gluconeogenesis and increased adipogenesis, both features of insulin insensitivity. IGF-I treatment was effective in increasing FOXO1, thereby suggesting that it could be considered as a potential treatment in CF patients possibly to prevent and treat cystic fibrosis-related diabetes.

## 1. Introduction

Cystic fibrosis (CF) is the most common life-threatening inherited recessive disease in the white population, and is caused by mutations in the CF transmembrane conductance regulator (CFTR) gene encoding a protein involved in the homeostasis of ions and other metabolites [1].

Cystic fibrosis-related diabetes (CFRD) is to date the major co-morbidity of CF [1,2,3], and its prevalence has dramatically increased in recent years. This condition is commonly ascribed to a progressive reduction in insulin secretion; however, the mechanisms underlying this progression are not understood, and a possible role of insulin resistance is debated [4,5,6,7].

We described previously that CF patients presented higher basal insulin concentrations compared with healthy controls, although insulin concentrations were within the normal range [8]. Furthermore, increased serum insulin peaks during oral glucose stimulation tests have been observed [9]. The findings possibly suggested reduced insulin sensitivity, which could be due to changes in insulin-receptor signal transduction, which have never been explored previously in CF patients. Moreover, it is very difficult to study insulin resistance clinically in these patients because of confounding factors as progressive insulin deficiency, malabsorption, and variable blood glucose concentrations [10].

Insulin action is initiated through the binding to and activation of its cell-surface receptor. This determines the activation of a signaling cascade that has been well characterized [11,12]. Briefly, insulin receptor activation is followed by insulin receptor substrates 1–4 phosphorylation/activation with subsequent activation of PI kinase, and Akt. Akt in turn is able to activate the winged helix forkhead (FOX) class of transcription factors. FOXO1 activates gluconeogenic genes in the liver and inhibits adipogenesis. Insulin is reported to reverse these effects through AKT/PKB, which phosphorylates FOXO1 on Ser256, inhibiting gluconeogenesis and activating adipogenesis [12]. Furthermore, FOXO1 is thought to play a key role in the development of type 2 diabetes mellitus [13,14]. The activation of the insulin receptor activates also the MAP kinases, ERK1 and 2, which are recognized as regulators of cell proliferation and differentiation [12].

Insulin resistance can be hypothesized as being related to the basic genetic defect, thus to CFTR malfunctioning, as well as to mechanisms depending on inflammation [8,9] and/or to other recently described mechanisms as reduced autophagy, and endoplasmic reticulum stress [15,16,17,18], CF is a well-recognized condition of chronic inflammation both at the cellular and systemic level. CF patients produce more TNFα than their counterparts [19]. We described also increased IL1, IL6 and TNFα serum concentrations in CF patients [8].

Recently, several cytokine-mediated mechanisms of insulin resistance have been described. For example, TNFα has been shown to block insulin action inducing serine/threonine phosphorylation of IRS1 [20,21], and studies in HepG2 cells and primary mouse hepatocytes have shown that exposure to IL6 is associated with reduced IRS1 phosphorylation, and reduced insulin-dependent activation of AKT [22].

Furthermore, the suppressors of cytokine signaling (SOCS), a family of negative regulators of cytokine signal transduction, are known to be induced by pro-inflammatory cytokines as IL6, and have been shown to directly interact with the insulin receptor (IR) or IRS1 inhibiting insulin signal transduction [23,24].

In recent years, β2 arrestins have been shown to be involved with regulation of insulin action and inflammatory signal pathway, particularly in type 2 diabetes [25], and thus could be of interest as regulators of insulin sensitivity in CF. More specifically, they can regulate IRS1 ubiquitination and degradation, preserve PI3K activation and subsequent phosphorylation of AKT, and scaffold to recruit MAP kinases and AKT to the IR [25,26]. Interestingly, Manson *et al*. reported recently increased β2 arrestin content in a CF *in vitro* model [27].

In humans, insulin sensitivity is regulated mainly by liver, adipose tissue, and skeletal muscle. A reduction in glucose uptake by the skeletal muscle is recognized to date as one of the principal mechanisms of insulin resistance, and intramyocellular lipid accumulation is thought to be one of the mechanisms involved [28,29].

A most promising therapy thus far for disorders of insulin resistance, due to defects in the IR, is rhIGF-I. Insulin-like growth factor 1 (IGF-I) binds mainly to the type I IGF-I receptor that shares the same post-receptor transducers as the IR, thus mediating insulin-like effects [30,31].

We aimed at studying insulin signal transduction in cystic fibrosis and wild-type cells to establish differences and investigate whether insulin sensitivity was altered in cystic fibrosis and related to CFTR loss of function.

We subsequently verified whether the observed changes were present in liver, white adipose tissue and skeletal muscle in a mouse model of CF, and finally verified the effects of IGF-I treatment in both the *in vitro* and *in vivo* models.

We provide evidence that insulin signal transduction in CF cells is impaired, is characterized mainly by reduced FOXO1 content, that these changes are related with CFTR loss of function, and that IGF-I is effective in increasing FOXO1 content both *in vitro*, and *in vivo* in skeletal muscle.

## 2. Results

### 2.1. Findings in CFBE41o- and 16HBE14o- Cells

#### 2.1.1. Insulin Receptor

**Figure 1:**
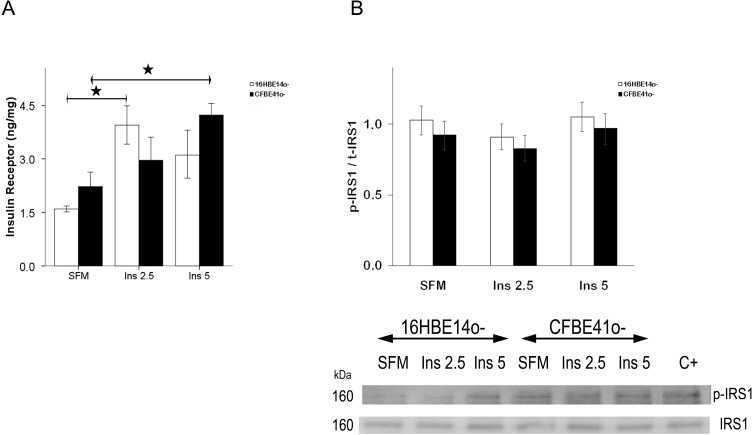
Total insulin receptor (IR) content (**A**), activated [Y941]/total insulin receptor substrate type 1 (IRS1) ratio (p-IRS1/t-IRS1) in normal and CF-affected cells (**B**). (**A**) IR content in CFBE41o- cells was similar to that in 16HBE14o- cells both in serum-free medium (SFM) and after treatment with insulin at 2.5 and 5 ng/mL. In 16HBE14o- cells, IR increased significantly with treatment; (**B**) IRS1 was quantified by Western immunoblotting after immunoprecipitation, p-IRS1/t-IRS1 content in CFBE41o- cells was similar to that in 16HBE14o- cells both in serum-free medium (SFM) and after treatment with insulin at 2.5 and 5 ng/mL. Beneath is a representative immunoblot showing tyrosine 941 phosphorylated IRS1 and total IRS1 in both cell types. C+: positive control. The bars represent the mean ± SEM of the ratios for four experiments in both cell types. ★ *p* < 0.05 SFM *vs*. insulin stimulation.

Total insulin receptor (IR) content was not different in control (16HBE14o-) and CFBE41o- cells at baseline and after stimulation with insulin (Figure 1A).

#### 2.1.2. Insulin Signal Transduction

Insulin receptor substrates (IRS) 1–4, downstream from the IR, are key molecules in signal transduction.

The activated/total IRS1 ratio was similar in the 16HBE14o- cells and in the CFBE41o- cells, and did not show significant changes after treatment with insulin (Figure 1B).

Tyrosine phosphorylation of IRS1 activates PI3Ks that play multiple roles in the regulation of cell survival, signaling, proliferation, migration and vesicle trafﬁcking. The p85α protein is the best-known regulatory subunit of PI3K.

**Figure 2:**
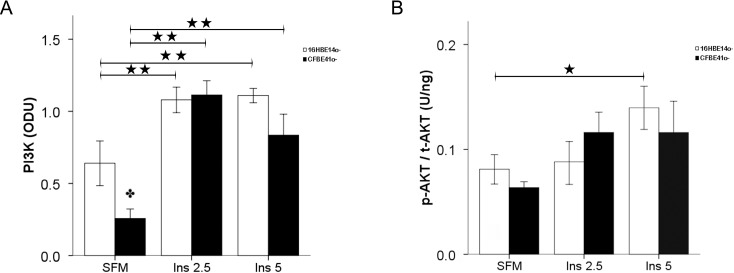
Phospho inositol kinase p85 subunit (p85 PI3K) content (**A**), and activated [S473]/total AKT ratio (p-AKT/t-AKT) (**B**) in CF-affected and normal cells. (**A**) p85 PI3K content, expressed in optic densitometry units (ODU), was lower in CFBE41o- cells in baseline conditions (SFM: serum-free medium). Insulin treatment increased significantly p85 PI3K content in both cell lines; (**B**) p-AKT/t-AKT ratio expressed in U/ng, was similar in both cell lines, and increased with insulin treatment. The bars represent the mean ± SEM of all experiments in both groups (*n* = 6). ★ *p* < 0.05 SFM *vs*. insulin treatment; ★★ *p* < 0.0001 SFM *vs*. insulin treatment; ✤ *p* < 0.05 CFBE41o- *vs*. 16HBE14o-.

At baseline, p85 PI3K content was significantly lower in the CFBE41o- cells but upon insulin stimulation increased significantly to levels similar to those in the normal cells. p85 PI3K content increased significantly from baseline in both cellular lines after treatment with insulin at both 2.5 and 5 ng/mL (Figure 2A).

AKT/PKB is a serine/threonine kinase that is a downstream target of PI3K signaling.

The activated/total AKT ratio increased in a dose dependent fashion in the 16HBE14o- cells with a significant increase from baseline at 5 ng/mL insulin stimulation. The baseline content was similar in the CFBE41o- cells and increased upon insulin stimulation with a maximal response at 2.5 ng/mL insulin concentration; however, this increase was not statistically significant (Figure 2B).

The winged helix forkhead (FOX) class of transcription factors is activated by AKT [12].

**Figure 3:**
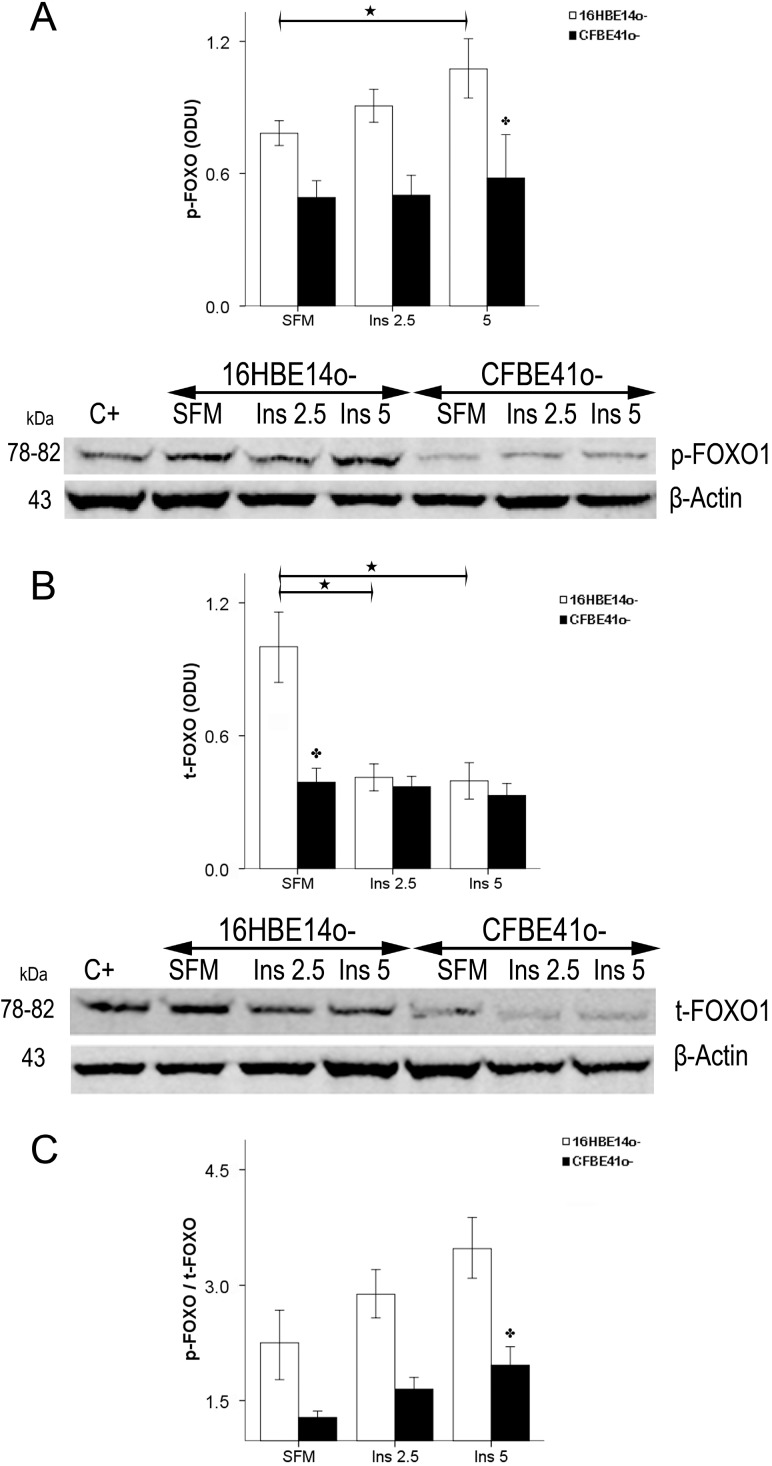
Phosphorylated [S256] FOXO1 (p-FOXO1) contents (**A**), total FOXO1 (t-FOXO1) contents (**B**), and p-FOXO1/t-FOXO1 ratio (**C**) in CF-affected and normal cells. (**A**) p-FOXO1, expressed in ODU was higher in 16HBE14o- cells and increased significantly with insulin stimulation, whereas no change was seen in CFBE41o- cells. Bars represent the mean ± SEM of all experiments in both groups (*n* = 6). SFM: serum-free medium. Beneath is a representative immunoblot showing p-FOXO1 in the different conditions in both cell lines. C+: positive control; (**B**) t-FOXO1 was significantly higher in serum-free medium (SFM) in 16HBE14o- with respect to CFBE41o- cells, and decreased significantly after insulin stimulation. In CFBE41o- cells the baseline content was lower and did not change with insulin stimulation. Bars represent the mean ± SEM of all experiments in both groups (*n* = 6). Beneath is a representative immunoblot showing p-FOXO1 in the different conditions in both cell lines. C+: positive control; (**C**) p-FOXO1/t-FOXO1 ratio increased with insulin stimulation in 16HBE14o- cells and was always more elevated than in CF cells. The bars represent the mean ± SEM of all experiments in both groups (*n* = 6). ★ *p* < 0.05 SFM *vs*. insulin treatment; ✤ *p* < 0.05 CFBE41o- *vs*. 16HBE14o-.

Phosphorylated FOXO1 content was always higher in the 16HBE14o- cells and increased significantly after stimulation with 5 ng/mL of insulin, whereas no change was observed in the CFBE41o- cells (Figure 3A). Total FOXO1 content was significantly higher at baseline in 16HBE14o- cells compared with CF cells, and decreased significantly in these cells after stimulation with insulin. In the CFBE41o- cells the baseline content was low and no change was evident after insulin stimulation (Figure 3B).

When the phosphorylated/total FOXO1 ratio was taken into consideration, a substantial difference between the two cell types was found. Whereas in the normal cells the ratio increased in a dose-dependent fashion upon insulin stimulation, concentrations in the CF cells were noticeably lower and showed only a slight increase after stimulation with insulin resulting significantly different from 16HBE14o- cells (Figure 3C).

β2 arrestins are capable of initiating distinct signals in their own right, and are recognized as AKT activators, suggesting that they can modify insulin signaling [25].

**Figure 4:**
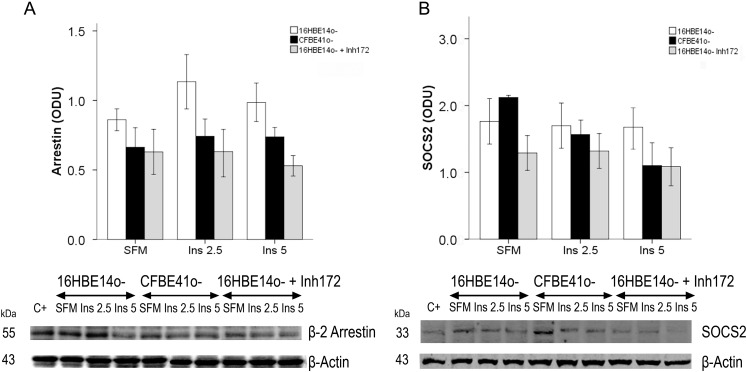
β2 arrestin (**A**), and suppressor of cytokine signaling (SOCS) 2 (**B**) in CF-affected and normal cells. (**A**) β2 arrestin, expressed in ODU, was lower, albeit not significantly, in CFBE41o- cells. Treatment of 16HBE14o- cells with a CFTR inhibitor (Inh172) did not determine any change in baseline conditions (serum-free medium—SFM), whereas in the presence of insulin, β2 arrestin content decreased noticeably; (**B**) Suppressor of cytokine signaling (SOCS) 2 was similar in both cell lines and did not change with insulin treatment. Treatment of 16HBE14o- cells with Inh172 lowered SOCS2 content to concentrations similar to those observed in CFBE41o- cells in the presence of insulin, although statistical significance was not attained. Bars represent the mean ± SEM of all experiments in both groups (*n* = 4). Beneath are representative Western immunoblots of samples from 16HBE14o-, CFBE41o- and 16HBE14o- treated with Inh172 as specified in methods. C+: positive control

β2 arrestin content was lower in the CF cells, although not significantly. No significant change was observed after insulin treatment in both cell lines (Figure 4A).

SOCS2 has been shown to directly interact with the IR or IRS1, inhibiting insulin signal transduction [23].

Similar SOCS2 contents were found in 16HBE14o- and in CFBE41o- cells with no significant change after stimulation with insulin in both cell lines. A small decrease was observed in the CFBE41o- cells after insulin stimulation (Figure 4B).

IR stimulation activates also ERK1/2 which controls mainly cell proliferation and differentiation [12].

Similar total ERK1/2 contents were observed in the two cell lines with no significant change after insulin stimulation, whereas activated ERK1/2 contents were higher in the CFBE41o- cells, and both decreased after stimulation with insulin at the highest concentration (5 ng/mL).

**Figure 5:**
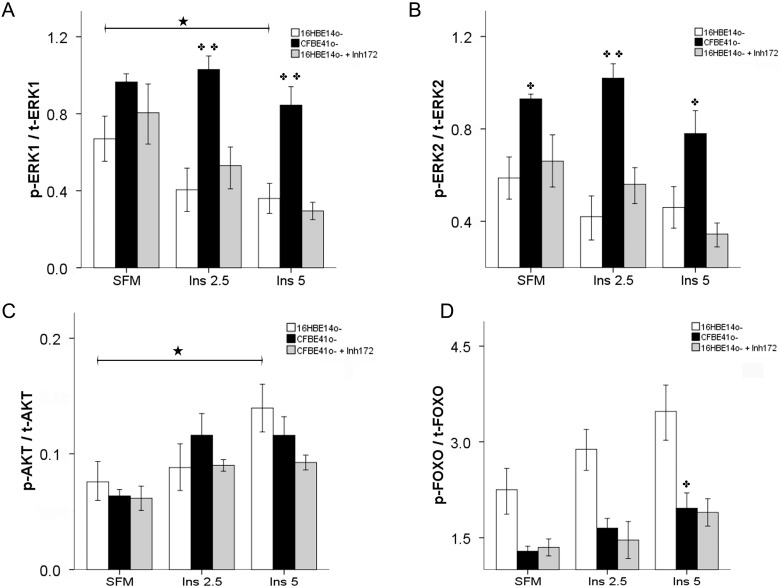
Activated [T202/Y204]/total ERK1 (p-ERK1/t-ERK1) ratio (**A**), activated [T185/Y187]/total ERK2 (p-ERK2/t-ERK2) ratio (**B**), activated [S473]/total AKT ratio (p-AKT/t-AKT) (**C**), and phosphorylated [S256]/total FOXO1 (p-FOXO1/t-FOXO1) ratio (**D**). (**A**) The p-ERK1/t-ERK1 ratio was similar in both cell lines in serum-free medium (SFM). After insulin stimulation, the ratio was significantly higher in the CFBE41o- cells compared with the normal cells. After treatment of 16HBE14o- cells with the CFTR inhibitor, no significant change was observed; (**B**) The p-ERK2/t-ERK2 ratio was significantly higher in the CFBE41o- cells compared with the normal cells in all conditions. CFTR inhibition in 16HBE14o- cells did not recreate the CF status; (**C**) Inh172 treatment of 16HBE14o- cells did not modify the p-AKT/t-AKT ratio in baseline conditions (SFM). After insulin stimulation at 5 ng/mL, the ratio was similar to the one found in CFBE41o- cells; (**D**) Inh172 treatment of 16HBE14o- cells lowered the p-FOXO1/t-FOXO1 ratio, in all conditions, to values similar to those found in the CFBE41o- cells. Bars represent the mean ± SEM of all experiments in both groups (*n* = 4). ✤ *p* < 0.05 CFBE41o- *vs*. 16HBE14o-; ✤✤ *p* < 0.0001 CFBE41o- *vs*. 16HBE14o-; ★ *p* < 0.05 SFM *vs*. insulin treatment.

The activated/total ERK1 ratio was significantly higher in the CFBE41o- cells compared with the normal cells and did not change after stimulation with insulin. In the 16HBE14o-cells the ratio decreased significantly when stimulated with insulin at 5 ng/mL (Figure 5A).

Findings for the activated/total ERK2 ratio were similar with a significantly increased ratio under all conditions in the CFBE41o- compared with the wild-type cells. The ratio did not decrease significantly in the 16HBE14o- after insulin stimulation (Figure 5B).

#### 2.1.3. Effect of Treatment of 16HBE14o- Cells with a Cystic Fibrosis Transmembrane Conductance Regulator (CFTR) Inhibitor and CFTR Gene Silencing

16HBE14o- were treated with CFTR inhibitor-172 in order to assess whether changes in insulin signaling between the two cell lines were dependent on CFTR function.

No significant change was observed for IRS1 and PI3K after treatment (data not shown).

In baseline conditions, the inhibitor did not modify significantly the activated/total AKT ratio but when the cells were incubated with insulin at 5 ng/mL, the ratio changed to concentrations similar to those found in the CFBE41o- cells (Figure 5C).

Interestingly, the phosphorylated/total FOXO1 ratio in the 16HBE14o- cells treated with the CFTR inh-172 decreased to very similar contents as those found in the CFBE41o- cells both in serum-free medium (SFM) and after treatment with insulin (Figure 5D).

Similarly, β2 arrestin content in 16HBE14o-cells, after treatment with the CFTR inhibitor, decreased both in baseline conditions and in the presence of insulin (Figure 4A).

Treatment of 16HBE14o- cells with the inhibitor lowered SOCS2 content to similar concentrations as in CFBE41o- cells in the presence of insulin, although statistical significance was not obtained (Figure 4B).

**Figure 6:**
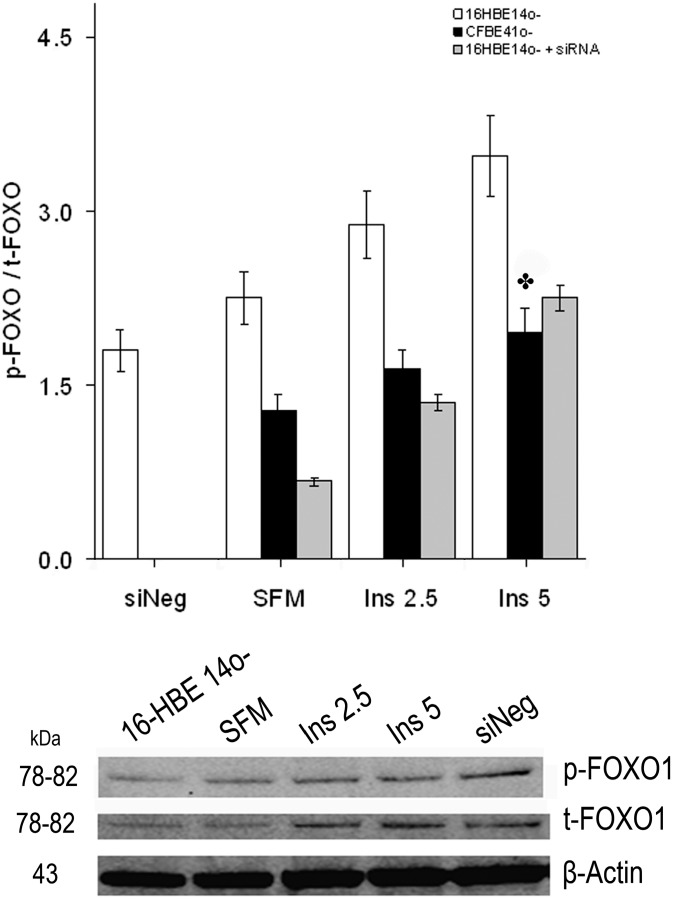
Phosphorylated [S256]/total FOXO1 (p-FOXO1/t-FOXO1) ratio after treatment of 16HBE14o- cells with the CFTR siRNA. CFTR siRNA treatment of 16HBE14o- cells lowered the ratio, to values similar to those found in the CFBE41o- cells. Beneath is a representative Western immunoblot of samples from both 16HBE14o-, and 16HBE14o- treated with CFTR siRNA as specified in methods. Bars represent the mean ± SEM of all experiments in both groups (*n* = 4). ✤ *p* < 0.05 CFBE41o- *vs*. 16HBE14o-.

CFTR gene silencing confirmed the findings described using the CFTR inhibitor 172. The phosphorylated/total FOXO1 ratio in the 16HBE14o- cells treated with CFTR siRNA decreased to very similar contents as those found in the CFBE41o- cells both in SFM and after treatment with insulin (Figure 6).

#### 2.1.4. Winged Helix Forkhead (FOX)O1 Changes after Treatment with IGF-I

**Figure 7:**
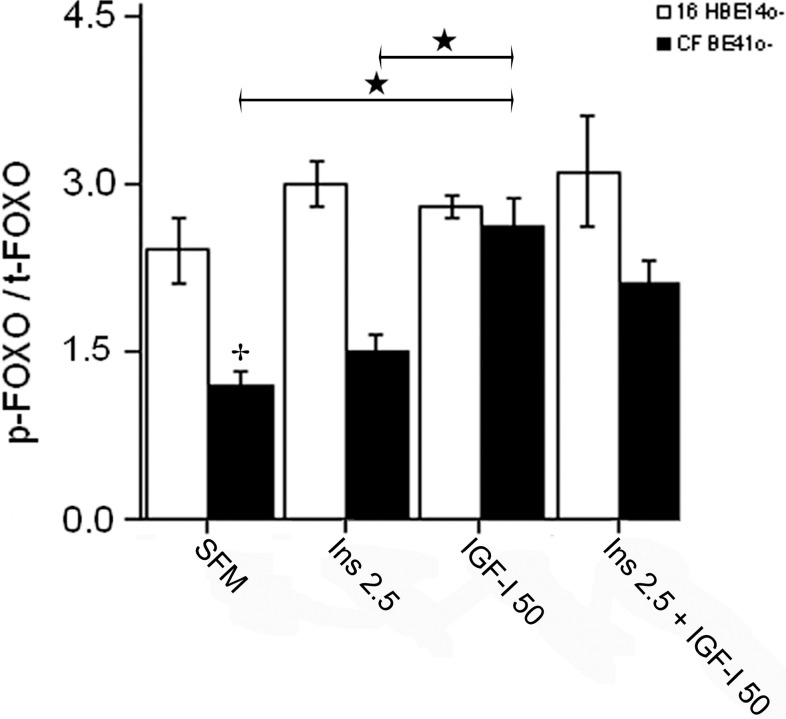
Changes in the phosphorylated [S256]/total FOXO1 (p-FOXO1/t-FOXO1) ratio after treatment of both 16HBE14o- and CFBE41o- cells with IGF-I (50 ng/mL). The two cell lines showed similar changes after IGF-I treatment alone, and in addition to insulin. The p-FOXO1/t-FOXO1 ratio increased in the CFBE41o- cells to a similar level as in 16HBE14o- cells. Bars represent the mean ± SEM of all experiments in both groups (*n* = 3). ★ *p* < 0.05 SFM and insulin treatment *vs*. insulin + IGF-I treatment; ✤ *p* < 0.05 CFBE41o- *vs*. 16HBE14o-.

The response to IGF-I treatment was similar in the two cell lines. In the CFBE41o- cells, IGF-I increased significantly the phosphorylated/total FOXO1 ratio from baseline, and with respect to insulin treatment alone. When IGF-I was added to insulin, the ratio increased by 40% with respect to baseline (Figure 7).

### 2.2. Findings in Normal and Cystic Fibrosis (CF)-Affected Mice

#### 2.2.1. Insulin Signal Transduction

**Figure 8:**
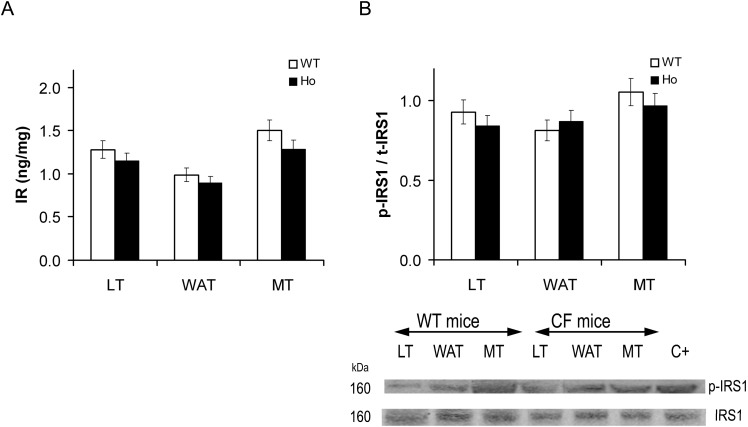
Insulin receptor content and p-IRS1/t-IRS1 ratio in mouse tissues. Total insulin receptor (IR) content (**A**), and activated [Y941]/total insulin receptor substrate type 1 (IRS1) ratio (p-IRS1/t-IRS1) (**B**) in 129/FVB CF-affected mice (Ho) and in their wild-type littermates (WT) in baseline condition in liver (LT), white adipose (WAT) and skeletal muscle tissues (MT). (**A**) IR was quantified using an ELISA kit and expressed in ng/mg of total protein content. IR content in CF mice was similar to WT mice in each tissue; (**B**) IRS1 was quantified by Western immunoblotting after immunoprecipitation, p-IRS1/t-IRS1 content in CF mice was similar to that in WT mice in all tissues. Beneath is a representative immunoblot showing tyrosine 941 phosphorylated IRS1 and total IRS1. C+: positive control. The bars represent the mean ± SEM.

In liver, white adipose tissue and vastus lateralis muscle, insulin receptor content was similar in CF-affected and in wild-type mice (Figure 8A).

The activated/total IRS1 ratio was similar in both mouse models, and did not show significant changes in the different tissues (Figure 8B).

**Figure 9:**
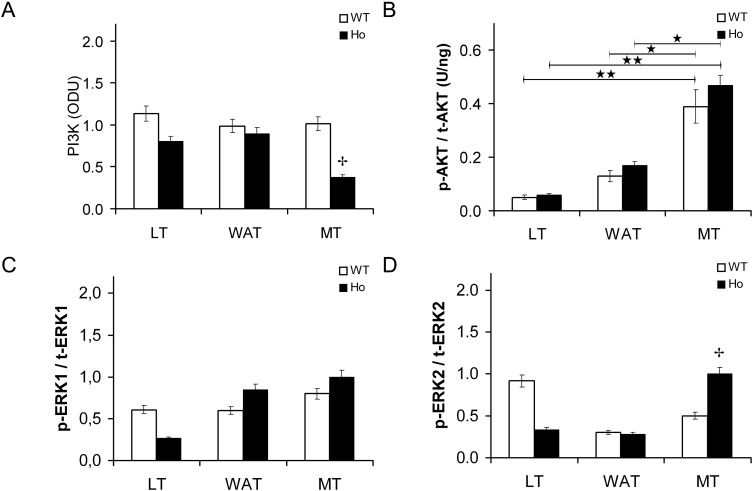
PI3K content, p-AKT/t-AKT, p-ERK1/t-ERK1 and p-ERK2/t-ERK2 ratios in mouse tissues. Phospho inositol kinase p85 subunit (p85 PI3K) content (**A**); activated [S473]/ total AKT ratio (p-AKT/t-AKT) (**B**); activated [T202/Y204]/total ERK1 (p-ERK1/t-ERK1) ratio (**C**); and activated [T185/Y187]/total ERK2 (p-ERK2/t-ERK2) ratio (**D**) in 129/FVB CF-affected mice (Ho) and in their wild-type littermates (WT) in baseline condition in liver (LT), white adipose (WAT) and skeletal muscle tissues (MT). (**A**) p85 PI3K content was expressed in optic densitometry units (ODU). p85 PI3K was lower in CF mice; (**B**) p-AKT/t-AKT ratio expressed in U/ng, was similar in both normal and CF mice, but higher in muscle tissue. The bars represent the mean ± SEM of all experiments in both groups. ★★ *p* < 0.01 LT *vs*. MT; ★ *p* < 0.05 WAT *vs*. MT; ✤ *p* < 0.05 wild-type (WT) *vs*. homozygote F508del (Ho) CF mouse.

p85 PI3K content was significantly lower in CF mice in muscle, whereas contents were similar to wild-type mice in both liver and adipose tissue (Figure 9A).

In liver, white adipose tissue and skeletal muscle, AKT content was similar in CF and in wild-type mice, as in the *in vitro* model. In the muscle, the AKT ratio was greater than in the other tissues (Figure 9B).

Similar total ERK1/2 contents were observed in the two mice models in all tissues. In muscle, the activated/total ERK2 ratio was greater in the CF-affected mice (Figure 9C,D).

**Figure 10:**
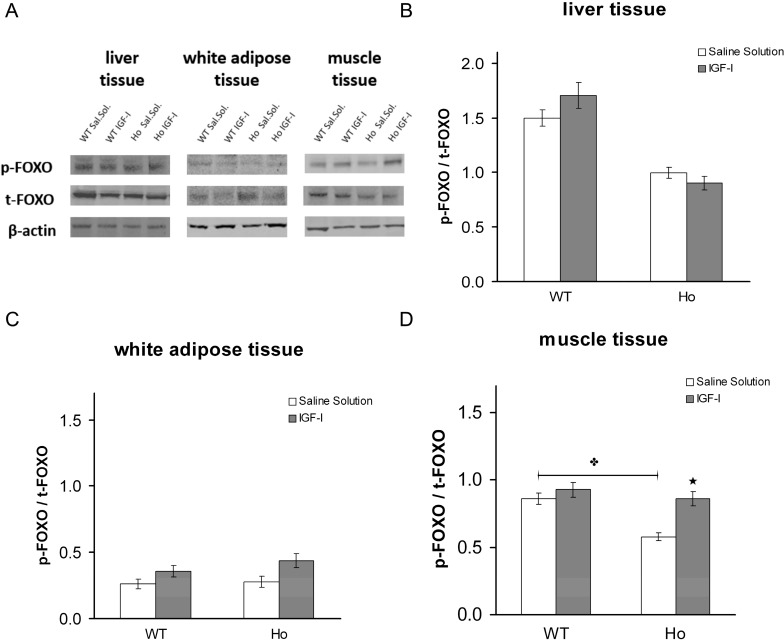
FOXO1 content in mouse tissues (**A**). Phosphorylated [S256]/total FOXO1 (p-FOXO1/t-FOXO1) ratio in liver (**B**); white adipose tissue (**C**); and skeletal muscle (**D**), in 129/FVB CF-affected mice (Ho) and in their wild-type littermates (WT) in baseline conditions and after treatment with rhIGF-I (4 µg/hg/day for 5 days). The p-FOXO1/t-FOXO1 ratio was similar in liver and white adipose tissue but was significantly different between normal and CF mice in skeletal muscle. IGF-I treatment increased the p-FOXO1/t-FOXO1 ratio in CF skeletal muscle to a value similar to that in WT. A non-significant increase was observed in white adipose tissue also. ✤ *p* < 0.05 wild-type (WT) mouse *vs*. homozygote F508del (Ho) CF mouse; ★ *p* < 0.05 saline solution *vs*. rhIGF-I treatment.

In muscle, the phosphorylated/total FOXO1 ratio was significantly lower in the CF-affected mice. In liver, no significant difference was detected between wild-type and CF-affected mice. The findings were similar in white adipose tissue also (Figure 10A–C).

#### 2.2.2. FOXO1 Changes after Treatment with rhIGF-I

Treatment with IGF-I significantly increased the activated/total FOXO1 ratio in muscle in the CF mice to contents similar to those found in wild-type mice (Figure 10A). An increase was observed after treatment also in white adipose tissue in both mouse models, and in liver in wild-type mice only (Figure 10B,C).

## 3. Discussion

The main findings of this study were normal total insulin receptor and p85 PI3K contents, a lesser increase in AKT, and lower and no change in FOXO1 contents in baseline conditions and after insulin stimulation in CF cells compared with normal cells. In addition, after stimulation with insulin, CF cells showed persistently elevated ERK1/2, reduced β2 arrestin content, and no change in SOCS2. Whereas the changes in FOXO1, β2 arrestin, and SOCS2 contents were dependent on CFTR loss of function, this was not apparent for AKT and ERK1/2. Findings were confirmed in the muscle of CF-affected mice as an *in vivo* model. Treatment with IGF-I was effective in increasing FOXO1 both *in vitro* and *in vivo*.

Insulin receptors are pleiotropic and control in all cell models cell proliferation, differentiation and apoptosis. To address studies on insulin sensitivity and signal transduction usually hepatocytes, adipocyte and myocyte cell models are used. However, these are as of yet unavailable with the common CFTR mutations and should be obtained from primary cultures of biopsies of patients, which would be unethical. Regulation of specific signaling is undoubtedly dependent on the specific cell type, and thus epithelial bronchial cells could have slightly different patterns of response to insulin. However, for cystic fibrosis, airway epithelial cells seemed to be an appropriate screening model to address for the first time a molecular study on insulin signal transduction. We further investigated and confirmed our *in vitro* findings in the pertinent tissues of a well-established animal model of cystic fibrosis [16].

Animals could be studied in baseline conditions only, corresponding to the fasting state, as insulin treatment results in the death of these animals because of their feeding habits and condition. Furthermore, at variance with humans, they do not live to develop CFRD.

As in other previously described conditions, we did not find any significant differences in the amount of insulin receptor [32], though we did in the signaling pathway downstream from the receptor.

Total and activated IRS1 contents were similar in both cell lines which did not suggest any significant change in insulin signaling in the two conditions at this level.

p85α PI3Kinase content was lower in baseline conditions in the CF cells, corresponding to the fasting state of an *in vivo* study. However, the response to insulin was preserved, thereby suggesting that PI3K is not a critical node.

The two cell types were not different in statistical terms, but the dose-dependent response of AKT to insulin was less evident in CF cells with no significant increase from baseline.

Changes in insulin signaling became apparent downstream from AKT. Activated AKT would have been expected to inactivate FOXO1 by phosphorylation [11]; however, this was not observed in CF cells. Interestingly, studies in KO mice have shown that AKT is not always an obligate intermediate in the pathway being analyzed [33]. In fact, it has been shown that FOXO1 can be regulated by acetylation, methylation, glycosylation, ubiquitination, and by other cofactors [33,34]. At present, however, it is not possible to put forward an explanation for these findings. Phosphorylation of FOXO1 blocks the gene transcription responsible for controlling gluconeogenesis and inhibiting adipogenesis [12,34]. These two mechanisms are therefore supposedly altered in CF. Reduced control of these two mechanisms could suggest intracellular lipid accumulation and persistent gluconeogenesis which could contribute to explaining why in clinical practice patients with CFRD are less prone to hypoglycemia when compared to subjects with type I diabetes mellitus. Furthermore, interestingly, our findings matched *in vivo* data previously described by Hardin *et al*., who described increased hepatic glucose production because of increased gluconeogenesis, and significantly lower suppression of gluconeogenesis by insulin, compared with controls [35].

Furthermore, the condition we describe in CF cells and in skeletal muscle of affected mice is similar to that described by Zhang *et al*. in FOXO1 knock-out mouse [36]. In this mouse model, a decrease in blood glucose concentrations, increased serum triglycerides and cholesterol concentrations, as well as hepatosteatosis were observed. Furthermore, the data of this study are consistent with the observations that CF subjects with abnormal glucose tolerance present de novo lipogenesis [37], and that misassembled mutant F508del CFTR alters cellular lipid trafficking [38].

CFTR activation and expression are regulated by the cAMP pathway, which is involved in cholesterol accumulation within cells. Interestingly, Manson *et al*. showed cholesterol accumulation in CF cells—although a different cell model to ours was used—and described a relationship with increased β2 arrestin protein expression. These findings [27] suggested a role for β2 arrestin in cellular responses in CF. Our findings were at variance with these results: lower β2 arrestin protein content in the CF cells with no significant increase after treatment with insulin. CFTR gene silencing and the use of the CFTR inhibitor in the wild-type cells determined a reduction in β2 arrestin which also suggests that the CFTR abnormality was involved in its regulation. It remains, however, unknown whether there is some other regulating factor down-regulating β2 arrestin in these cells upon insulin stimulation. Interestingly, β2 arrestin- KO primary mouse embryonic fibroblasts have been reported to show increased Akt phosphorylation and ERK1/2 activation [38] that are also consistent with our findings. β2 arrestin could be also a regulator of ERK1/2 as previously discussed [39]. Furthermore, ERK1 has been shown to be required for adipogenesis *in vitro* and *in vivo* in adipocytes [40]. Although there is no definite explanation at present, the increase in these MAP Kinases is a consistent finding of interest, and recently an explanation has been provided through the activation of the protein kinase tumor progression locus 2, a key molecule in relaying inflammatory stimuli to ERK1/2 [41].

SOCS2 was not different in normal and CF cells, and the total content decreased in response to insulin at variance with the wild-type cells. SOCS2 double KO mice exhibit increased hepatic triglyceride secretion with improved insulin sensitivity, and increased NFkB activity [24], thus suggesting that in CF cells the lower SOCS2 contents could be a compensatory mechanism to favor insulin sensitivity, and could be due also to cross-talk among different receptors.

The effect of treatment of normal cells with the CFTR inhibitor, and the gene silencing experiments, suggested that the changes observed were mainly due to CFTR loss of function, and thus specific for CF. The findings related to FOXO1 content in the tissues of the CF-affected and wild-type mice is of great interest as it would suggest differences already in the fasting state, and, in particular, would locate in the muscle the main mechanisms related with insulin insensitivity, whereas changes were not so evident in the liver and in adipose tissue. These findings are compatible with data in the literature, thereby supporting a close link between intracellular lipid accumulation and insulin insensitivity in skeletal muscle [30].

This is of relevance to future research on cystic fibrosis.

Finally, CFRD is also well known to be due to a reduction in insulin secretion that manifests over time [4,9,42]. Interestingly, in human beta cells, it has been shown recently that FOXO1 is a core component in adaptation to metabolic stress, and impaired FOXO1 activation would cause or exacerbate diabetes [43]. FOXO1 content has been shown to be implicated also with lipid-induced beta-cell apoptosis and glucose-stimulated insulin secretion impairment [14,44,45]. Further investigation would be warranted to verify these aspects in beta-cells from CF patients also that could explain impaired insulin secretion besides insulin insensitivity.

IGF-I treatment has been previously used in humans to overcome very serious conditions of insulin resistance [30,31]. *In vitro* the (2560)/total FOXO1 ratio was normalized by IGF-I treatment, and the datum was confirmed in the muscle and less but also, in white adipose tissue of affected mice, supporting the hypothesis that it could be a possible candidate for the treatment and/or prevention of CFRD. Interestingly, recent data have shown that *in vitro* IGF-I increases CFTR expression and progression to the plasma membrane [46], suggesting that some of the observed effects could be related to improved CFTR expression. IGFI is the main GH-dependent growth factor in humans; however, it became unavailable for treatment for a period (European Medicines Agency 25 April 2013 EMA/250270/2013), and its availability remains scarce. Interestingly, trials with the use of GH in CF patients have shown that it is capable of increasing IGFI, improving muscle efficiency, in addition to improving insulin sensitivity [47,48,49,50,51], and could therefore represent a potential additional treatment.

In conclusion, we are the first to provide evidence of a molecular defect in the insulin signaling pathway in CF, compatible with insulin insensitivity. The data in CF cells showed reduced FOXO1 activity in the fasting state, and reduced FOXO1 inactivation in the presence of insulin. Changes were also present in the *in vivo* model, particularly in skeletal muscle. Finally, IGF-I treatment was effective in increasing FOXO1 activity, thereby suggesting that it could be considered as a potential treatment in CF patients, possibly to prevent and treat CFRD.

## 4. Experimental Section

### 4.1. Cell Lines, Cultures, Insulin and IGF-I Treatments

Two airway epithelial cell lines: CFBE41o-, homozygous for the F508del mutation, (derived from a bronchial isolate of a CF patient homozygous for the F508del CFTR mutation), and 16HBE14o- (derived from normal bronchus) as non-CF control, were used. CFBE41o- and 16HBE14o- cells were a kind gift from Dr. Dieter C. Gruenert. All cell lines were immortalized with the pSVori-plasmid [51].

Cells were grown as previously described [52,53]. Before stimulation, the cells were washed and starved in serum-free medium (SFM) for 24 h.

Cells were stimulated with insulin (PROSPEC –Tany technogene Ltd., East Brumswich, NJ, USA) at 2.5 and 5 ng/mL as maximum response was observed at these concentrations. Cell lysates were obtained after 6-, 12-, 24- and 48-h incubation with the stimuli or SFM. Data in the text are reported at 24 h where the best protein response was obtained.

IGF-I was acquired from PeproTech (London, UK) and used at 50 ng/mL for 24 h incubation in serum-free medium, as previously reported [54]. All experiments were repeated at least 3 times for each cell line.

Paraformaldehyde-fixed cell sections were analyzed for evidence of protein cytocheratin 7 using immunohistochemistry to check for appropriate cell differentiation (data not shown). The CFTR gene was sequenced in both cell lines and confirmed the genotype of both cell lines (data not shown).

Plating efficiency was determined at cell attachment and had to be greater than 80%.

### 4.2. Total Protein Content

Lysates were obtained using modified RIPA buffer (Tris-HCL: 50 mM, pH 7.4, NP-40: 1%, Na-deoxycolate 0.25%, NaCl 150 mM, EDTA 1 mM, and PMSF 1 mM, Aprotinin, leupeptin and pepstatin 1 μg/mL each one, Na_3_VO_4_ 1 mM, NaF 1 mM as protease inhibitors), and stored at –80 °C until assayed. The total protein content was determined using the “microassay Bio-Rad” protocol (Bio-Rad Lab, Munchen, Germany). Briefly, a standard curve was prepared using bovine albumin (Bio-Rad Lab, Hercules, CA, USA). The dye was added to each sample and standard and the absorbance was read at 595 nm using Victor 4x multilabel reader (Perkin Elmer, Waltham, MA, USA).

### 4.3. Protein Assays in Lysates

Total IR content was assayed using a specific research kit by Invitrogen Life technologies (Grand Island, NY, USA). The intra-assay coefficient of variation (CV) was 5.4%, and the inter-assay CV was 8.2%. The sensitivity of the method was <0.15 ng/mL.

Total AKT was assayed using a specific research kit by Invitrogen Life Technologies (Grand Island, NY, USA). The intra-assay CV was 7.7%, and the inter-assay CV 9.3%. The sensitivity of the method was <0.1 ng/mL.

Activated AKT [pS473] was quantified using a research kit (Invitrogen Life technologies, Grand Island, NY, USA) specific for phosphorylation at serine residue 473. The intra-assay CV was 6.9%, and the inter-assay CV was 8.3%. The sensitivity of the method was 0.8 U/mL.

Concentrations were normalized per mg of total protein content in the lysates, and phosphorylated substrates were normalized relative to the total amount of specific protein.

### 4.4. Immunoprecipitation (IP)

Immunoprecipitation was carried out using same amount of whole cell lysate, 2.5 μg of specific antibody and 1.5 mg of Dynabeads, according to the manufacturer’s instructions (Life Technologies, Grand Island, NY, USA).

### 4.5. Western Immunoblotting

Equal aliquots of lysates (40 μg total protein) were subjected to electrophoresis on 4%–12% gradient NuPAGE Novex Bis-Tris gels and 3%–8% gradient NuPAGE Novex Bis-Tris gels, as appropriate (Life Technologies, Grand Island, NY, USA). The proteins were then transferred to nitrocellulose membranes (Amersham Biosciences Ltd., GE Healthcare Europe GmbH, Glattbrugg, Swizerland).

For insulin receptor signal transduction, the following antibodies were used: anti-IRS1, anti-phospho IRS1 [Y941] and [S307] (Millipore corporation, Tamecula, CA, USA); anti-ERK1/2, anti phospho-[T202-Y204/T182-Y187] ERK1/2 and anti SOCS2 (Santa Cruz Biotechnology Inc, Santa Cruz, CA, USA); anti p85 PI3K, anti-FOXO1, anti-phospho-FOXO1 [S256] and anti β2 arrestin purchased from Cell Signaling Technology (Denver, MA, USA).

Anti βactin (Cell Signaling Technology; Denver, MA, USA) was used to normalize the optic densities of all molecules. The IRS-1 peptide (Millipore corporation, Tamecula, CA, USA), and an internal lysate were used as positive controls as previously shown (32).

Anti-rabbit IRDye 680/800 or anti-mouse IRDye 680/800 (LI-COR, Lincoln, NE, USA) antibodies were used as appropriate, as second antibodies. Detection was obtained by Odyssey Infrared Imaging System (LI-COR, Lincoln, NE, USA). The intensity of the bands was quantified using Image Studio 2.0 (LI-COR inc, Lincoln, NE, USA) for the Odyssey Infrared Imaging System and expressed as optical densitometry units (ODU).

### 4.6. CFTR Inhibition and Gene Silencing

Only normal cells (16HBE14o- cells) were treated with the CFTR inhibitor (CFTR inh 172) to recreate the CF condition [55,56].

Cells were treated with either DMSO (Sigma, St. Louis, MO, USA) as vehicle control or 20 μM CFTR inh 172 (Sigma, St. Louis, MO, USA) prepared in DMSO and diluted from a 1:1000 stock. Media was replenished every 6 h for 48 h for the protein studies.

No toxicity from the treatment with the CFTR inhibitor was observed using trypan blue exclusion assay.

To reduce the amount of endogenous CFTR, 16HBE14o- cells were transfected with siRNA against human CFTR (FlexiTube GeneSolution GS1080 for CFTR). All reagents for silencing were obtained from Qiagen (Valencia, CA, USA). We used a pooled solution of 4 siRNA (target sequence: 5'TCGATATATTACTGTCCACAA3', 5'ATCGCGATTTATCTAGGCATA3', 5'CTCGAAAGTTATGATTATTGA3' and 5'ATGGCCAACTCTCGAAAGTTA3'). Transfection was carried out by using HiPerFect transfection reagent and 10 nM siRNA per well, according to the manufacturer’s instructions. Negative control siRNA was use as control. Silencing was monitored at the mRNA level using QuantiFast Probe assay double probe Kits (β actin housekeeping gene) for one-step real-time RT-PCR. Gene silencing was approximately 55% (data not shown).

### 4.7. Animal Models and IGF-I Treatment

Young adult female CF mice homozygous for the F508del-CFTR mutation [57] in the 129/FVB outbred background (Cftrtm1EUR, F508del, FVB/129) were obtained from Bob Scholte, Erasmus Medical Center Rotterdam, The Netherlands (CF coordinated action program EU FP6 LSHM-CT-2005-018932). These studies and procedures were approved by the local Ethics Committee for Animal Welfare (IACUC No. 382) and conformed to the European Community regulations for animal use in research (CEE no. 86/609). Young adult CF mice and their wild-type (wt) littermates were housed in static isolator cages at the animal care-specific pathogen-free facility of Charles River (Calco, Italy), stored and bred at the European Institute for Research in Cystic Fibrosis (I.E.R.F.C.), San Raffaele Institute, Milan, Italy as previously described [16] following the recommendations of the Federation of European Laboratory Animal Science Associations [58]. Mice were 3 to 4 months of age and weighed between 20 and 30 grams. To prevent CF intestinal obstruction, Movicol (55.24 g/L; Norgine, Heverlee, Belgium) was administered in acidified drinking water.

Skeletal muscle from the vastus lateralis muscle, liver, and white adipose tissues were immediately rinsed in ice-cold sterile saline solution, split into two parts, and snap-frozen in liquid nitrogen. Tissues were lysed in modified RIPA buffer within seven days, and lysates were then subjected to electrophoresis and Western immunoblotting. Eight mice in each group were sacrificed and analyzed.

Mice were treated with 4 µg/hg/day (mecasermine) of rhIGF-I for 5 days, a kind gift from Ipsen Pharma, (Boulogne-Billancourt, France).

### 4.8. Statistical Analysis

Standard statistical analysis was performed using the statistical package SPSS 18.0 as appropriate.

Data were presented as mean ± SEM and analyzed by General Linear Model (GLM), which subsumes traditional regression, ANOVA, and ANCOVA including repeated measures analysis. A *P* value <0.05 was considered statistically significant.

## 5. Conclusions

This paper provides the first evidence of a molecular defect in the insulin signaling pathway in CF, compatible with insulin insensitivity. The data in CF cells showed reduced FOXO1 activity in the fasting state, and reduced FOXO1 inactivation in the presence of insulin. Changes were also present in the *in vivo* model, specifically in skeletal muscle. Finally, IGF-I treatment was effective in increasing FOXO1 activity, thereby suggesting that it could be considered as a potential treatment in CF patients, possibly to prevent and treat CFRD.

## Supplementary Materials

Supplementary materials can be found at: http://www.mdpi.com/1422-0067/15/10/18000/s1.

## Supplementary Files

Supplementary File 1
