# Supplementary material for: FOXO1 Content Is Reduced in Cystic Fibrosis and Increases with IGF-I Treatment
Source: Int J Mol Sci. 2014 Oct 8;15(10):18000–22. doi: 10.3390/ijms151018000 (PMC4227201; doi:10.3390/ijms151018000)
Supplement: Supplementary File 1 [file ijms-15-18000-s001.pdf]

Supplementary Information

Insulin signaling in cystic fibrosis (CF)

Insulin binds to its receptor activating phosphorylation of insulin receptor substrates 1–4 (IRS) on specific thyrosine residues. These activate phosphatidylinositol 3 kinase (PI3K) in the plasmalemma, which in turn determines the activation/phosphorylation of protein kinase B (AKT) in the cytosol. The p85α protein is the best-known regulatory subunit of PI3K. AKT regulates the expression of gluconeogenic and lipogenic enzymes by controlling the activity of the winged helix or forkhead (FOXO) class of transcription factors. FOXO1, in the fasting state (blue and red lines marked with (1)), activates gluconeogenic genes and inhibits adipogenesis. Phosphorylation of FOXO1, in the presence of insulin (blue and red lines signed with (2)), blocks gene transcription, thereby inducing gluconeogenesis and activating adipogenesis. Both total and activated FOXO1 contents, and the activated/total FOXO1 ratio, are reduced in CF, Intracellular lipid accumulation (reduced inhibition of adipogenesis) and persistent gluconeogenesis are thus expected, and have been previously shown. By using a Cystic Fibrosis CFTR inhibitor and siRNA, changes in FOXO1 were related with CFTR loss of function. Increased ERK1/2, reduced β2 arrestin, and unchanged SOCS-2 contents were also shown *in vitro.* Findings were confirmed *in vivo*, in a CF mouse model, in the main tissues known to regulate insulin sensitivity (skeletal muscle, liver and white fat tissue).
